# Comparing the Effect of Phenytoin Syrup and Triamcinolone Acetonide Ointment on Aphthous Ulcers in Patients with Behcet’s Syndrome

**Published:** 2012-02-01

**Authors:** M M Fani, H Ebrahimi, S Pourshahidi, E Aflaki, S Shafiee Sarvestani

**Affiliations:** 1 Department of Oral Medicine, Dental School, Shiraz University of Medical Sciences, Shiraz, Iran; 2 Department of Rheumatology, Shiraz University of Medical Sciences, Shiraz, Iran; 3 Dentist, Shiraz University of Medical Sciences, Shiraz, Iran

**Keywords:** Phenytoin, Triamcinolone Acetonide, Aphthous ulcer, Behcet’s syndrome

## Abstract

**Background:**

Recurrent aphthous stomatitis (RAS) appears to be the most common type of oral ulcers. The lesion is usually self limited but its painful presentation results in some difficulties. Therefore, an efficient therapeutic strategy is required and currently existing therapies seem to be inadequate because of its unclear etiology. Here the therapeutic effect of triamcinolone acetonide ointment as a relatively expensive medication has been compared with phenytoin syrup on aphthous ulcers in patients with Behcet’s syndrome.

**Methods:**

Thirty out of 60 our patients with Behcet’s syndrome were randomly treated by phenytoin syrup and the remaining were advised to use 0.1% triamcinolone acetonide ointment. After a week, they were visited again to determine the status of aphthous ulcers.

**Result:**

Positive response in the triamcinolone acetonide group and phenytoin group was 86.7% and 53.3%, respectively.

**Conclusion:**

The effectiveness of triamcinolone acetonide ointment was more than phenytoin on aphthous ulcers in patients with Behcet’s syndrome.

## Introduction

Recurrent aphthous stomatitis (RAS) appears to be the most common type of oral ulcers. Typically it is presented as self limited ulcers affecting nonkeratinized oral mucosa.[1] The first lesions often appear when the patient is a child.[2] It has a higher prevalence in younger adults and the incidence and severity would decrease with age.[3][4][5][6][7] No gender predilection has been detected.[3]

Minor, major and herpetiform aphthous ulcers are the subdivisions. Minor ones, seen in 80% of patients[8][9][10] are less than 10 mm in diameter. The round or ovoid ulcers with a pseudo membranous base surrounded by erythema, often appear on labial or buccal mucosa, heal within 7-10 days and leave no scar.[1][2][8][11]

Major aphthous ulcers are presented as larger and deeper ulcers, mostly seen on soft palate, tonsils, tongue or pharynx, heal within several weeks and usually leave scar. Herpetiform aphthous usually present as ulcers of 1-3 mm in diameter and occur in groups. They heal within 1-4 weeks.[1][2][8][12][13][14][15][16][17]

The cause of RAS is still unknown, although there are many promoting and exacerbating factors such as positive family history, trauma, nutritional deficiency (iron, vitamin B12, folate), food hypersensitivity, immune disturbance, smoking cessation and psychological stress.[1][2][8][12][13][14][15][16] RAS generally is not associated with systemic disorders but aphthous like ulcers may be the presentation of some disorders such as Behcet’s and Reiter’s syndrome.[2][17]

Although the lesion is usually self limited, its painful presentation leads to difficulty in eating, speaking and swallowing and decreased quality of life. Patients may suffer from its high frequency, too.[1] So an efficient therapeutic strategy is needed and because of its unclear etiology, current available therapies seem to be inadequate.[1][13]

The therapeutic strategies for minor aphthous lesions include concurrent oral hygiene, coating agents, antiseptics, antibiotics, hormones, topical anesthesia and steroids. These methods mostly relieve symptoms and accelerate healing but systemic ones, although effective, have side effects. Therefore, topical therapies are the first choice.[8] Here, we are to compare the therapeutic effect of triamcinolone acetonide ointment as a relatively expensive and not easily available medication in Iran, with phenytoin syrup on aphthous ulcers in patients with Behcet’s syndrome.

Triamcinolone acetonide is a fluoride synthetic corticosteroid. Its cream (0.1%) and ointment (0.1%) forms are available for topical use. The absorption rate varies from 1% in palms and knee to 36% in face, eyelash and genital area. Its absorption increases via damaged, inflammed or dressed skin. Steroids may have systemic absorption via oral mucosa.[18]

Metabolism of triamcinolone after topical application is dermal. The small amount which may enter systemic circulation is metabolized in liver. Topical application’s side effects are burning, itching, irritation, dryness, folliculitis, hirsutism, hyperpigmentation, perioral dermatitis, contact allergic dermatitis, secondary infections and atrophy.[18]

Phenytoin was first made in 1908 and its anticonvalescent effect was detected in 1938. The most common side effect of phenytoin is gingival hyperplasia. These effects were reported in 1939.[19] Dill and Iacopino reported a case of skin thickening due to long term application of phenytoin. Thickening occurred in face and Talon which revealed the proliferative effect of the medication on connective tissue cells in whole body.[20]

In an animal study, the angiogenesis effect of phenytoin was shown. It also led to decreased inflammation and bacterial colonies, necrosis and proliferation of fibroblasts.[21] Phenytoin affected epithelium via keratinocyte growth factors and their receptors which may have a significant role on wound healing. Ghapanchi et al. applied phenytoin powder on wound secondary to flap and reported less edema and inflammation and more granulation tissue and fibrosis. It also led to less pain and burning sensation and accelerated wound healing clinically.[22] Phenytoin can be used as syrup (125 mg/5 ml), ampule (250 mg/5 ml), cream (1%) and capsule (50 and 100 mg). The classic form of topical application is suspension (30 mg/5 ml).[23]

In this study, the therapeutic effect of triamcinolone acetonide ointment as a relatively expensive medication was compared with phenytoin syrup on aphthous ulcers in patients with Behcet’s syndrome.

## Materials and Methods

Sixty patients with Behcet’s Syndrome who had oral aphthous lesions were assessed for eligibility to participate in this trial (IRCT code: IRCT201107036920N2). It was their first visit and did not have any medication for the disease. All were visited by a rheumatologist and a dentist. They all answered a questionnaire about demographic data, the age of Behcet’s syndrome onset, the most affected oral site with the aphthous ulcers, duration of ulcers and severity of pain and burning. Then the dentist visited them and recorded the site of aphthous lesions, their size and number.

Thirty patients were randomly selected and treated by phenytoin syrup and other 30 ones were advised to use 0.1% triamcinolone acetonide ointment. Those in triamcinolone group applied the ointment three times a day on the lesions and had been advised not to have water or meal till 30 minutes. Those in phenytoin group used 2 teaspoon of syrup in half a glass of warm water as mouthwash for 4-5 minutes, three times a day and had been advised not to have water or meal till 30 minutes. After a week, they were visited again to determine the status of aphthous ulcers.

In this study, the group treating by triamcinolone was named “ T” and the other named “P”. The statistical analysis was performed using SPSS software (Version 14.0, Chicago, IL, USA). We used Fisher’s Exact test and Independent t test to compare positive response of the groups and the mean of patients’ age, respectively.

## Results

There were 8 men and 22 women in each group (Table 1). The mean age in “T” group was 35.47+8.85 years and in “P” group was 38.77+9.4 years (Table 1). So the mean age and gender in these two groups were almost equal and could not interfere in the results (p>0.05). Among 30 patients in “T” group, 4 of them (13.3%) did not respond to the medication and 26 of them (86.7%) had a positive response. In “p” group, 14 of them (46.7%) did not respond to the medication and 16 of them (53.3%) had a positive response. Statistical tests revealed that triamcinolone ointment had a better therapeutic effect on aphthous lesions in patients with Behcet’s syndrome (p=0.01).

**Table 1:** The demographic characteristics of patients treated with phenytoin (P group) and triamcinolone acetonide (T group).^a^

	**Sex**		**Age (years)**		
	**Men**	**Women**	**Min**	**Max**	**Mean+SD**
P group	8 (26.7%)	22 (73.3%)	17	65	38.77±9.4
T group	8 (26.7%)	22 (73.3%)	15	57	35.47±8.85

^a^ p=0.167

## Discussion

Aphthous ulcer is the chief complain of a significant percentage of patients visited by physicians or dentists. It sometimes interferes with patients’ activities and decreases their quality of life because of severe pain and burning. Some specialists have immunomodulative strategy. Brown and Bott applied combination of azathioprine and dexamethasone on aphthous ulcers, topically and had a good result.[24] Miles and Steven revealed that triamcinolone acetonide is a useful medication for aphthous lesion.[24] This is similar to our findings which shows that corticosteroids are effective in treatment of aphthous lesions.

In an animal study, Ghapanchi and colleagues applied phenytoin powder on wound secondary to flap and reported less edema and inflammation and more granulation tissue and fibrosis. It also led to less pain and burning sensation and clinically an accelerated wound healing.22 In our study, healing of most aphthous ulcers with phenytoin application resembled their results.

There was not any study to compare the effect of these tow medications. In our study, the positive response to triamcinolone acetonide (86.7%) was more than phenytoin (53.3%) (p<0.05). So it is not logical to replace phenytoin instead of triamcinolone; although triamcinolone ointment is more expensive and less available. Triamcinolone acetonide ointment was shown to be more effective than phenytoin on aphthous ulcers in patients with Behcet’s syndrome.
